# Neurotoxin Treatment Injection Time for Upper Facial Lines

**DOI:** 10.1111/jocd.71044

**Published:** 2026-07-09

**Authors:** Julia Garcia, Elena Dimitrijevic, Jeremy B. Green

**Affiliations:** ^1^ AbbVie Irvine California USA; ^2^ Skin Research Institute & Skin Associates of South Florida Coral Gables Florida USA

To the Editor,

Injectable neuromodulators are a popular treatment choice among patients seeking to improve their skin appearance by reducing wrinkles formed from repeated muscle activity, including forehead, glabellar, and crow's feet upper facial lines (UFLs) [1]. Botulinum neurotoxin type A (BoNTA), including onabotulinumtoxinA, is a group of safe, effective, and noninvasive treatments that require less recovery time than surgical procedures [1, 2, 3]. However, the time it takes to inject BoNTA is not well documented; indeed, much of the available literature focuses on the time from injection to effect onset or effect duration rather than injection time itself [4, 5]. Clinical guidance from the American Society of Plastic Surgeons broadly state that these injections “usually take less than 15 minutes” [6]. Quantifying the average injection time may influence whether a patient starts or a provider recommends treatment. In this preliminary evaluation of injection practices, investigators surveyed healthcare professionals (HCPs) to quantify the time required to administer onabotulinumtoxinA injections for the simultaneous treatment of the 3 types of UFLs and explore reasons for treating the 3 UFLs in a single visit.

Between December 16, 2021 and January 10, 2022, a target population of 300 injectors in the United States completed a self‐administered online survey. Respondents were sampled from online physician panels maintained by Harris Insights and Analytics LLC (The Harris Poll). Respondents were licensed plastic surgeons (*n* = 100), dermatologists (*n* = 100), and other aesthetic HCPs (*n* = 100) (e.g., primary care physicians, nurse practitioners). All respondents were required to have ≥ 2 years in practice, spend ≥ 75% of their time conducting patient care, conduct aesthetic treatments and/or consultations with ≥ 50 patients per month, and directly treat ≥ 20 patients per month with onabotulinumtoxinA injections for all 3 UFLs in a single visit.

Respondents provided informed consent and demographic information and answered procedural questions regarding onabotulinumtoxinA injections. They estimated the average time it takes to perform the injections for all 3 UFLs, with injection time defined as the time from withdrawing reconstituted solution from a vial into a syringe to administering the last injection.

Approximately half of the respondents were male (54.0%). Practice settings included single‐specialty partnerships/groups (46.0%), solo practices (33.0%), and multispecialty partnerships/groups (20.0%) (Table 1). Plastic surgeons and dermatologists had a higher average number of years in practice versus respondents in the aesthetic HCP group (16 vs. 11 years). Nearly two thirds (63.8%) of patients treated were aged 35–64 years.

**TABLE 1:** Respondent demographics.

	Plastic surgeons (*n* = 100)	Dermatologists (*n* = 100)	Aesthetic HCPs^a^ (*n* = 100)	Overall (*N* = 300)
Age, mean (SD), years	50.8 (9.86)	45.6 (9.75)	40.1 (9.04)	45.5 (10.48)
Age, *n* (%), years				
18–34	1 (1.0)	12 (12.0)	32 (32.0)	45 (15.0)
35–44	32 (32.0)	40 (40.0)	40 (40.0)	112 (37.3)
45–54	33 (33.0)	31 (31.0)	20 (20.0)	84 (28.0)
55–64	24 (24.0)	10 (10.0)	6 (6.0)	40 (13.3)
65+	10 (10.0)	7 (7.0)	2 (2.0)	19 (6.3)
Gender, *n* (%)				
Female	23 (23.0)	35 (35.0)	78 (78.0)	136 (45.3)
Male	77 (77.0)	64 (64.0)	21 (21.0)	162 (54.0)
Nonbinary or gender nonconforming	0 (0.00)	1 (1.0)	1 (1.0)	2 (0.67)
Years in practice, mean (SD)	17.6 (9.62)	14.2 (10.11)	17.1 (7.65)	16.0 (9.83)
Years in practice, *n* (%)				
2–10	27 (27.0)	46 (46.0)	52 (52.0)	125 (41.7)
≥ 11	73 (73.0)	54 (54.0)	48 (48.0)	175 (58.3)
Age group of patients who received injections, %				
18–24 years	6.4	5.3	6.4	6.0
25–35 years	15.7	16.4	18.7	16.9
35–44 years	29.5	31.3	31.9	30.9
45–64 years	33.5	33.7	31.6	32.9
≥ 65 years	14.9	13.4	11.5	13.3
Patients seen per month, mean (SD)	162.5 (87.81)	218.1 (179.41)	165.4 (114.22)	182.0 (134.85)
Patients seen per month by appointment type, mean (SD)				
Cosmetic consultations or treatments	162.5 (87.81)	218.1 (179.41)	165.4 (114.22)	182.0 (134.85)
Cosmetic neuromodulator injections	74.6 (53.85)	112.6 (92.79)	99.8 (76.11)	95.7 (77.32)
Treatment of 3 UFLs in 1 visit	47.1 (34.57)	72.2 (73.96)	67.4 (57.44)	62.2 (58.47)
Practice setting, *n* (%)				
Dermatology office/practice	3 (3.0)	92 (92.0)	39 (39.0)	134 (44.7)
Plastic surgery office/practice	80 (80.0)	1 (1.0)	13 (13.0)	94 (31.3)
Multispecialty office/practice	14 (14.0)	4 (4.0)	30 (30.0)	48 (16.0)
Medi‐spa	2 (2.0)	3 (3.0)	15 (15.0)	20 (6.7)
Other	1 (1.0)	0 (0.0)	3 (3.0)	4 (1.3)
Description of practice setting, *n* (%)				
Single‐specialty partnership or group	41 (41.0)	59 (59.0)	39 (39.0)	139 (46.3)
Solo practice	39 (39.0)	26 (26.0)	34 (34.0)	99 (33.0)
Multispecialty partnership or group	20 (20.0)	15 (15.0)	25 (25.0)	60 (20.0)
Chain	0 (0.0)	0 (0.0)	2 (2.0)	2 (0.7)
Region of practice, *n* (%)				
South	38 (38.0)	32 (32.0)	38 (38.0)	108 (36.0)
West	20 (20.0)	29 (29.0)	21 (21.0)	70 (23.3)
Northeast	21 (21.0)	21 (21.0)	23 (23.0)	65 (21.7)
Midwest	21 (21.0)	18 (18.0)	18 (18.0)	57 (19.0)

*Note:* *n*(%) in the table denotes the number (percentage) of respondents.

Abbreviations: HCP, healthcare professional; N/A, not available; SD, standard deviation; UFL, upper facial line.

^a^ Aesthetic HCPs included primary care physicians, nurse practitioners, licensed practical nurses, registered nurses, and physician assistants.

Respondents estimated a mean injection time of 10.0 min (standard deviation [SD], 7.85 min; 95% confidence interval [95% CI], 9.1–10.9) for the administration of all 3 UFLs (Figure 1); median injection time was also 10.0 min. All respondents endorsed the simultaneous treatment of the 3 UFLs; notably, providers reported that patients requested single‐visit treatment on their own (58.0%) more often than relying on the injector's recommendations (42.0%). Respondents reported recommending treating all 3 UFLs simultaneously due to “better results overall” (79.0%), patient preference (65.0%), anatomical considerations (i.e., treatment of glabellar and forehead lines together) (61.0%), and convenience (51.0%). Further, most respondents agreed that it is important for providers to explain the benefits of treating all 3 UFLs simultaneously (96.0%) and that treating all 3 UFLs in 1 visit results in improved patient outcomes (94.0%). Finally, many respondents (60.0%) believed that provider recommendations are a major influence on patients' decisions to seek injections, followed by patient testimonials (37.0%), social media (35.0%), promotions (24.0%), and advertisements (16.0%).

**FIGURE 1:**
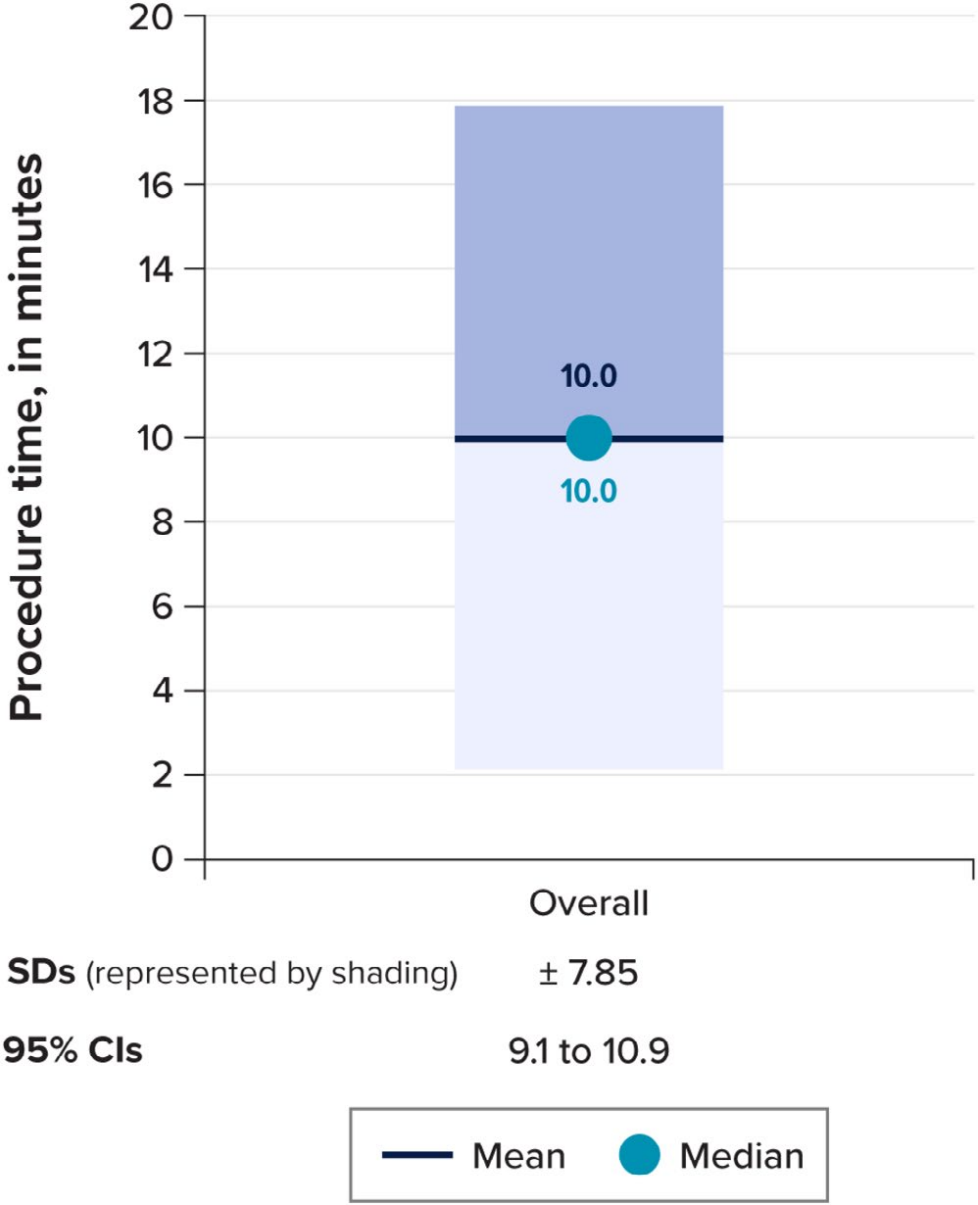
Injection Time. CI, confidence interval; SD, standard deviation. Represents the overall sample of plastic surgeons, dermatologists, and aesthetic HCPs.

Limitations inherent to survey research should be considered, including the potential for recall bias and social desirability bias; as such, providers may be more likely to underreport the amount of time it takes to simultaneously treat all 3 UFLs. As the primary outcome of this study was total injection time from drawing up the solution to actual injection, patients may experience a shorter injection time in practice if onabotulinumtoxinA is drawn outside of the patient room.

This study quantified the average injection time for onabotulinumtoxinA for the simultaneous treatment of 3 UFLs. With an average injection time of 10 min for all 3 UFLs, BoNTA treatment is a relatively quick procedure. Understanding the time required to receive onabotulinumtoxinA injections may be useful in shared decision‐making discussions between patients and providers, increase patient likelihood of initiating or continuing treatment, and increase provider likelihood of recommending BoNTA for the treatment of UFLs. These practical procedural details may assist injectors and patients when determining whether cosmetic neuromodulator procedures, specifically BoNTA, are appropriate. Future studies may expand upon these results to explore factors that contribute to differences in injection times (e.g., injector experience) and how injection time is associated with patient satisfaction with BoNTA treatment.

## Funding

Allergan Aesthetics, an AbbVie Company, funded this study and participated in the design, research, analysis, data collection, interpretation of data, and review and approval of the publication. RTI Health Solutions, an independent nonprofit research organization, received funding under a contract with AbbVie to provide publication support in the form of manuscript writing, styling, and submission.

## Ethics Statement

This study protocol was deemed exempt from Institutional Review Board (IRB) oversight by the Advarra IRB per US Department of Health and Human Services regulations (45 CFR 46.104(d) (2)). All participants provided informed consent prior to completing the survey.

## Conflicts of Interest

No honoraria or payments were made for authorship. Financial arrangements of the authors with companies whose products may be related to the present report are listed as declared by the authors: J.B.G. is an investigator, speaker, and trainer for Allergan Aesthetics, an AbbVie Company. J.K.G. and E.D. are full‐time employees of AbbVie.

## Data Availability

The data can be requested by any qualified researchers who engage in rigorous, independent, scientific research and will be provided following review and approval of a research proposal, Statistical Analysis Plan, and execution of a Data Sharing Agreement. Data requests can be submitted at any time after approval in the US and Europe and after acceptance of this manuscript for publication. The data will be accessible for 12 months, with possible extensions considered. For more information on the process or to submit a request, visit the provided link (https://vivli.org/ourmember/abbvie/), then select “Home.”
